# Correction: Preda et al. The Efficacy of Powered Oscillating Heads vs. Powered Sonic Action Heads Toothbrushes to Maintain Periodontal and Peri-Implant Health: A Narrative Review. *Int. J. Environ. Res. Public Health* 2021, *18*, 1468

**DOI:** 10.3390/ijerph191912389

**Published:** 2022-09-29

**Authors:** Camilla Preda, Andrea Butera, Silvia Pelle, Eleonora Pautasso, Alessandro Chiesa, Francesca Esposito, Giacomo Oldoini, Andrea Scribante, Anna Maria Genovesi, Saverio Cosola

**Affiliations:** 1 Tuscan Stomatologic Institute, Versilia General Hospital, 55041 Lido di Camaiore, Italy; camilla.preda@unipv.it (C.P.); silviapelledh@gmail.com (S.P.); pautasso.e@gmail.com (E.P.); francesca.esposito@unipv.it (F.E.); giacomo.oldoini88@gmail.com (G.O.); anmgen@tiscali.it (A.M.G.); s.cosola@hotmail.it (S.C.); 2 Study Center for Multidisciplinary Regenerative Research, Guglielmo Marconi University, 00100 Rome, Italy; alessandro.chiesa@unipv.it; 3 Department of Clinical, Surgical, Diagnostic an Paediatric Sciences University of Pavia, 27100 Pavia, Italy; andrea.scribante@unipv.it; 4 Department of Dentistry, Unicamillus International Medical University, 00100 Rome, Italy; 5 Department of Stomatology, University of Valencia, 46001 Valencia, Spain

There was an error in the original article [1]. “In Figures 2–4, respectively, the distribution of the mean Gingival Index, Bleeding Score, and Plaque Index for the oscillating toothbrushes (first line/dark) and sonic ones (second line/clear) were reported” should be “In Figures 2–4, respectively, the distribution of the mean Gingival Index, Bleeding Score, and Plaque Index for the sonic toothbrushes (first line/dark) and oscillating ones (second line/clear) were reported” (“oscillating” instead of “sonic” and “sonic” instead of “oscillating”. A correction has been made to the Results section, page 6.

There was another error in the original article [1]. The sentence was not clear. A correction has been made to the Discussion section, page 13.

Incorrected paragraph: “The studies selected for this review had diverse study designs with follow-ups, ranging from two weeks to 24 weeks. The toothbrushes used and the outcomes measured also varied in each study. Therefore, the clinical results of both oscillating toothbrushes and sonic toothbrushes were compared throughout the percentages of changing from the baseline.”

Corrected paragraph: “The studies selected for this review had diverse study designs with follow-ups, ranging from two weeks to 24 weeks. The toothbrushes used and the outcomes measured also varied in each study. Therefore, the clinical results of both oscillating toothbrushes and sonic toothbrushes were compared throughout the percentages of changing from the baseline, respectively corresponding to the clear (ORHs) and dark (SHAs) line.”

There was another error in the original article [1]. The sentence was not clear. A correction has been made to the Discussion section, page 14.

Incorrected paragraph: “Notably, in the Schmickler et al. investigation, SAH assigned subjects experienced a 19.18% Gingival Index mean reduction, versus 3.39% obtained with ORHs toothbrushes.”

Corrected paragraph: “Schmickler et al. reported that SAH-assigned subjects experienced a 20.34% (23.73–3.19%) Gingival Index mean reduction (dark line) versus the 8.2% (19.18–10.98%) obtained with ORH toothbrushes (clear line).”

The authors apologize for any inconvenience caused and state that the scientific conclusions are unaffected. The original article has been updated.
